# Inspiratory oscillatory flow with a portable ventilator: a bench study

**DOI:** 10.1186/cc3531

**Published:** 2005-05-17

**Authors:** Guenther E Frank, Helmut Trimmel, Robert D Fitzgerald

**Affiliations:** 1 Director, Department of Anaesthesiology and Intensive Care, General Hospital Barmherzige Brüder Eisenstadt, Austria; 2 Director, Department of Anaesthesiology and Intensive Care, General Hospital Wiener Neustadt, Austria; 3 Director, Ludwig Boltzmann Institute for Economics of Medicine in Anesthesia and Intensive Care, Vienna, Austria

## Abstract

**Introduction:**

We observed an oscillatory flow while ventilating critically ill patients with the Dräger Oxylog 3000™ transport ventilator during interhospital transfer. The phenomenon occurred in paediatric patients or in adult patients with severe airway obstruction ventilated in the pressure-regulated or pressure-controlled mode. As this had not been described previously, we conducted a bench study to investigate the phenomenon.

**Methods:**

An Oxylog 3000™ intensive care unit ventilator and a Dräger Medical Evita-4 NeoFlow™ intensive care unit ventilator were connected to a Dräger Medical LS800™ lung simulator. Data were registered by a Datex-S5™ Monitor with a D-fend™ flow and pressure sensor, and were analysed with a laptop using S5-Collect™ software. Clinical conditions were simulated using various ventilatory modes, using various ventilator settings, using different filters and endotracheal tubes, and by changing the resistance and compliance. Data were recorded for 258 combinations of patient factors and respirator settings to detect thresholds for the occurrence of the phenomenon and methods to overcome it.

**Results:**

Under conditions with high resistance in pressure-regulated ventilation with the Oxylog 3000™, an oscillatory flow during inspiration produced rapid changes of the airway pressure. The phenomenon resulted in a jerky inspiration with high peak airway pressures, higher than those set on the ventilator. Reducing the inspiratory flow velocity was effective to terminate the phenomenon, but resulted in reduced tidal volumes.

**Conclusion:**

Oscillatory flow with potentially harmful effects may occur during ventilation with the Dräger Oxylog 3000™, especially in conditions with high resistance such as small airways in children (endotracheal tube internal diameter <6 mm) or severe obstructive lung diseases or airway diseases in adult patients.

## Introduction

Transport ventilators, until recently, were simple flow interrupters with constant flow, allowing only a few parameters to be changed and with no, or only very limited, alarm and monitoring functions. These devices are still in use by emergency services and for mechanical ventilation of critically ill patients during intrahospital and interhospital transport [1]. The development of transport ventilators in recent years has introduced flow and pressure monitoring and has enabled the setting of positive end expiratory pressure (PEEP), inspiration to expiration ratio, and pressure limits. This made it possible to use these devices not only in emergency medicine, but also for transport of critically ill patients with severe lung injury. Nevertheless, the continuation of sophisticated mechanical ventilation during transport of critically ill patients with acute lung failure often still required the use of an intensive care ventilator. The higher weight, the higher power consumption, and the additional need for compressed air, as well as the larger dimensions, make transport with conventional intensive care ventilators more complicated and trouble-prone [2-6].

The Oxylog 3000™ transport ventilator (Dräger Medical, Best, The Netherlands) combines the properties of a modern intensive care ventilator with the advantages of a compact transport ventilator, such as low weight, small dimensions, and low power consumption. The main innovation of the Oxylog 3000™ is the possibility to use pressure-controlled, pressure-limited ventilation and pressure support.

We therefore used the Oxylog 3000™ routinely since 2002 for interhospital transfer, but have detected an undesired oscillatory flow during inspiration in paediatric patients and in adult patients with airway obstruction. The phenomenon occurred during pressure-regulated or pressure-limited ventilation and was characterised by four to eight rapid changes in flow velocity. The peak airway pressure exceeded the previously set pressure values and the phenomenon was accompanied by a reduction in minute ventilation. The phenomenon clinically impressed with a staccato-like breathing sound, similar to jet ventilation, and it was sometimes possible to detect a jerky thorax excursion during inspiration, even if the patient received neuromuscular blocking agents. Following these experiences, we conducted a bench study simulating different ventilator settings and respiratory conditions with the Oxylog 3000™ in comparison with a standard intensive care respirator – the Evita-4 NeoFlow™ (Dräger Medical).

## Materials and methods

The Oxylog 3000™ allows the setting of all common modes of ventilation used in critically ill patients, including intermittent positive pressure ventilation (IPPV), biphasic intermittent positive airway pressure (BIPAP), which can be used as pressure controlled ventilation, assisted spontaneous breathing (ASB), which is equivalent to pressure support ventilation, continuous positive airway pressure, synchronised intermittent mandatory ventilation, and noninvasive ventilation with leakage compensation. Flow is generated and regulated by means of four magnetic valves. Only oxygen is required as the gas supply since ambient air is added for adjustment of the F_I_O_2 _from 40% to 100% by means of a Venturi valve. Further adjustable parameters are the I:E ratio, the tidal volume (V_T_), the respiratory rate, the pressure limit, the PEEP, the ramp of inspiratory flow in BIPAP and ASB (slow, standard, fast), and the flow trigger. A flow sensor is positioned close to the patient. The pressure curve, the flow curve and the following parameters can be shown on the display of the Oxylog 3000™: peak P_aw_, mean P_aw_, plateau P_aw_, PEEP, expiratory V_T_, respiratory rate and expiratory minute volume.

**Figure 1:**
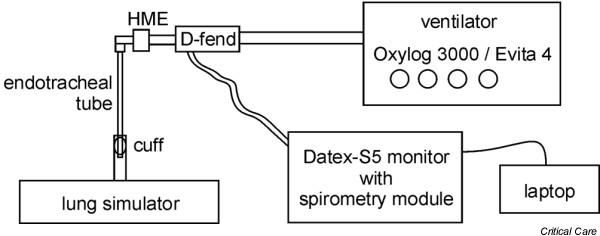
Scheme for the experimental set-up. HME, heat and moisture exchange filter.

The setting of the bench study is demonstrated in Fig. 1. The ventilators, the lung simulator, and the test laboratory were provided by Dräger Medical™ (Vienna, Austria). Prior to performing the tests, all apparatus were checked for faults and correct function. Reusable tubing was used for both ventilators. A spirometry sensor, a heat and moisture exchange filter (DAR Tyco™ Healthcare, Mansfield, MA, USA), and an endotracheal tube were connected between the ventilator and the lung simulator in an airtight manner by means of the inflated cuff, which was checked for leakage prior to the measurements. The spirometry was performed with a Datex-S5™ monitor (Datex-Ohmeda™, Helsinki, Finland) with D-fend™ sensors in different sizes (paediatric, adult). This Datex-S5™ monitor is routinely used in anaesthesia and intensive care medicine, and uses a double line sensor inserted between the heat and moisture exchange filter and the tubing. The Datex-S5™ monitor was connected to a laptop using specific software (S5-Collect™, Datex-Ohmeda™, Helsinki, Finland) to store and analyse the measured data.

The stepwise changed parameters and respirator settings of the 258 tests are summarised in Table 1. The special combinations of patient factors and respirator settings were chosen to detect thresholds for the occurrence of the phenomenon. All measurements in the IPPV mode were taken with a pressure limit of 20 cmH_2_O.

The following parameters were recorded in all tests. From the display of the ventilators, the respiratory rate, the V_T_, the peak P_aw_, the F_I_O_2_, warnings and alarms were read and recorded manually. The S5-Collect™ software stored the data measured by the spirometry module, including the peak P_aw_, the mean P_aw_, the plateau P_aw_, the PEEP, the intrinsic PEEP, the inspiratory V_T_, the expiratory V_T_, the compliance, the resistance, the duration of inspiration and the I:E ratio.

**Figure 2:**
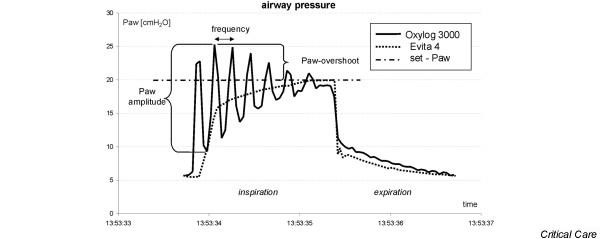
Airway pressure oscillation (Paw). Typical oscillatory Paw curve of the Oxylog 3000™ (solid line) in comparison with the Evita-4 NeoFlow™ (broken line). Settings: endotracheal tube internal diameter, 5 mm; biphasic intermittent positive airway pressure, 20/5 cmH_2_O; respiratory rate, 20/min; inspiration to expiration time ratio, 1:1; ramp, fast. The maximal amplitude of the pressure oscillation is the airway pressure amplitude (Paw-amplitude). Paw-overshoot, airway pressure overshoot.

**Figure 3:**
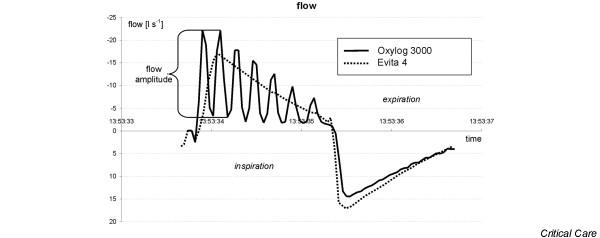
Flow curve oscillation. Typical oscillatory flow curve of the Oxylog 3000™ (solid line) in comparison with the Evita-4 NeoFlow™ (broken line), with the same settings as Fig. 1. The minimal flow was calculated by dividing the expiratory tidal volume, measured by the Oxylog 3000™, through the time of inspiration.

For each measurement the P_aw _curve and the flow curve of six respiratory cycles and the trend data of all measured parameters were stored in a separate file. The curves were quantitatively analysed with Microsoft Excel™ to evaluate the duration of the oscillations as a percentage of the inspiration time, the frequency of the oscillations, the amplitudes of the pressure oscillation (P_aw-ampl_), the amplitude of the flow oscillation, and the maximal inspiratory flow (Figs 2 and 3). If the peak P_aw _was higher than the set inspiratory pressure in the pressure-regulated modes or higher than the set pressure limit in IPPV, the difference between these values was calculated and stored as the airway pressure overshoot (Fig. 2). The magnitude of the P_aw-ampl _and the amount of the airway pressure overshoot were used to describe the severity of the phenomenon.

Selected tests were performed with both the Oxylog 3000™ and the Evita-4 NeoFlow™ to validate the measurements and to compare ventilation with the two ventilators under exactly the same conditions. One hundred and ninety-eight tests were performed with the Oxylog 3000™ and 60 comparative measurements were taken with the Evita-4 NeoFlow™, producing a total of 258 tests. Differences in the peak P_aw_, the mean P_aw_, and the expiratory V_T _between the two ventilators were calculated.

The maximal inspiratory flow velocity seemed to have an important influence on the occurrence and severity of the phenomenon, and we therefore calculated the ratio of the maximal flows between the Oxylog 3000™ and the Evita-4 NeoFlow™.

### Statistics

As indicated by Kolmogorov-Smirnov tests, the data showed deviations from a normal distribution, thus precluding the computation of parametric descriptive and inference statistics. Results are thus presented as the median with the interquartile range, minimum and maximum, and the Spearman rank correlations were computed. The Mann-Whitney U test was used to examine the differences between the Oxylog 3000™ and the Evita-4 NeoFlow™. *P *≤ 0.05 was considered significant.

## Results

No oscillatory flow was detected in any test using the Evita-4 NeoFlow™ respirator. Overall with the Oxylog 3000™, an oscillatory flow was detected in 90% of all respective measurements. The phenomenon was seen in the pressure-regulated modes BIPAP, ASB, continuous positive airway pressure, and in pressure-limited IPPV. No significant difference in the expiratory V_T _was detected when comparing the Oxylog 3000™ measurements with oscillatory inspiratory flow with the corresponding Evita-4 NeoFlow™ tests. Nevertheless, the oscillations resulted in significant higher peak and mean P_aw _on comparing the two ventilators (Table 2).

The duration and the shape of the pressure oscillations depended on the mode, on the ramp, and on whether a leakage was simulated. In general the curve oscillated around the normally seen P_aw _curve, which could be observed in comparison with the P_aw _curve of the Evita-4 NeoFlow™ measurements.

It was possible to measure the frequency of the oscillations in 153 tests with a median frequency of 5 Hz(interquartile range, 1.25 Hz; minimum, 2.78 Hz; maximum, 12.5 Hz). There was a trend to lower frequencies of the oscillations when the phenomenon was more severe. In the tests with a measured frequency of 4.17 Hz the median P_aw-ampl _was 23.8 cmH_2_O, with a frequency of 5 Hz the median was 15.8 cmH_2_O, and with a frequency of 12.5 Hz the median P_aw-ampl _was 6.9 cmH_2_O.

Concerning the severity of the phenomenon, the median values of P_aw-ampl _and the amount of the airway pressure overshoot are summarised in Table 3.

**Figure 4:**
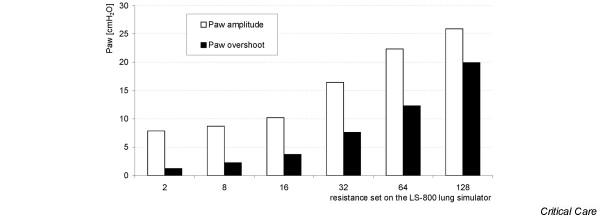
Influence of the test lung – resistance. Stepwise increase of the resistance on the LS800™ lung simulator resulted in an increase of the amplitude of the airway pressure oscillations (Paw-amplitude) as well as in an increase in the airway pressure overshoot (Paw-overshoot), defined as peak airway pressure minus the upper pressure limit.

**Figure 5:**
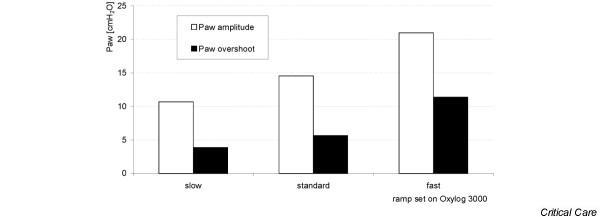
Influence of the steepness of the ramp on the phenomenon. A stepwise increase of the ramp, set on the Oxylog 3000™, resulted in an increase of the amplitude of the airway pressure oscillations (Paw-amplitude) as well as in an increase in the airway pressure overshoot (Paw-overshoot), defined as the peak airway pressure minus the upper pressure limit.

The severity of the phenomenon increased when the resistance on the LS800™ lung simulator (Dräger Medical, Best, The Netherlands) was increased (Fig. 4). The steepness of the ramp, set on the Oxylog 3000™, correlated positively with the severity of the oscillations (Fig. 5).

Changing the compliance on the LS800™ lung simulator did not have any influence on the occurrence and severity of the phenomenon. This was also true for the respiratory rate, the PEEP, the time of inspiration, and the I:E ratio. An intrinsic PEEP was detected in 126 of the Oxylog 3000™ measurements but did not show any correlation to the phenomenon. No influence of the set F_I_O_2 _on the occurrence of the phenomenon was seen. The phenomenon also occurred with 100% oxygen when the Venturi valve was not active.

**Figure 6:**
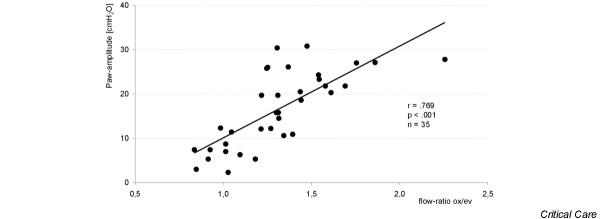
Peak inspiratory flow. Correlation between the airway pressure amplitude (Paw-amplitude) and the ratio of the peak inspiratory flow with the Oxylog 3000™ to the peak inspiratory flow with the Evita-4 NeoFlow™ (flow-ratio ox/ev). Only measurements in the biphasic intermittent positive airway pressure or the intermittent positive pressure ventilation modes without leakage and without single spikes in the flow curve were included.

We investigated the differences between the maximal inspiratory flow velocities generated by the two ventilators. The flow generated by the Oxylog 3000™ was usually higher than that with the Evita-4 NeoFlow™. The ratio of the maximal flows between the Oxylog 3000™ and the Evita-4 NeoFlow™ correlated well to the severity of the phenomenon, expressed as P_aw-ampl _(Fig. 6).

The oscillations almost exclusively occurred during inspiration. In general, the flow pattern of the expirations was no different compared with the corresponding Evita-4 NeoFlow™ measurements. An oscillatory flow during expiration was only seen in some of the measurements with simulated leakage, when the ventilator had to generate a flow directed to the test lung during expiration to maintain the PEEP. The oscillations during expiration had a P_aw-ampl _of 5 cmH_2_O and a frequency of 4.17 Hz. In comparison with equivalent measurements without leakage there seemed to be an attenuating effect of the leakage on the severity of the inspiratory oscillations.

## Discussion

While no oscillatory flow could be detected with the Evita-4 NeoFlow™, the phenomenon was found in a high percentage of tests with the Oxylog 3000™. We have to point out, however, that this high percentage is due to the setting of our tests, chosen to induce and investigate the phenomenon. Fifty per cent of the tests were taken with small endotracheal tubes, and the phenomenon was surprisingly seen in all tests with endotracheal tubes with internal diameter ≤ 6 mm in pressure-regulated modes, irrespective of the test lung conditions. Only five tests with endotracheal tubes of internal diameter ≤ 6 mm showed no oscillatory flow, and all of them were taken in the IPPV mode without reaching the pressure limit (constant flow).

Two parameters had an impact on the occurrence and severity of the phenomenon: the resistance, and the peak velocity of the inspiratory flow. The latter is influenced by the ramp set on the Oxylog 3000™ and by the interaction of test lung conditions and ventilator settings. The ratio of the maximal inspiratory flow measured with the Oxylog 3000™ to the maximal flow measured with the Evita-4 NeoFlow™ is another way to describe an inappropriate high flow at the beginning of the inspiration, and the value correlated well to the severity of the phenomenon.

The following hypothesis was made to explain why an oscillatory flow occurs under conditions with high resistance and high initial flow. The maximal inspiratory flow, reached during pressure-regulated ventilation, mainly depends on the airway resistance. The initial flow generated by the Oxylog 3000™ in the BIPAP and ASB modes depends on the ramp, and in the mode of pressure-limited IPPV it depends on the set V_T_. The flow, initially generated by the Oxylog 3000™, sometimes is much higher than the flow that can traverse the resistance set on the test lung. After initiation of the inspiration with an inappropriate high flow, the inspiratory pressure or the pressure limit (set on the Oxylog 3000™) is reached very rapidly and the flow is downregulated or stopped by the Oxylog 3000™. This does not occur rapidly enough and the P_aw _exceeds the target value. The pressure drops after the reduction or interruption of the flow and the flow is generated too late and too high again. Thus the pressure oscillates around the desired level. The oscillations are a result of rapid changes between exceedingly high and low flow velocities. The feedback mechanism between measured P_aw _and flow generation does not work rapidly enough or sensitively enough to smoothly adjust the inspiratory flow to an appropriate level.

We might explain the expiratory oscillation, seen in some measurements with a simulated leakage, by the fact that during expiration the leakage has to be compensated by a flow delivered by the Oxylog 3000™ to maintain the PEEP.

The results of the bench study predict a high probability for the phenomenon to occure in paediatric patients with narrow airways. This is exactly what we have seen in clinical practice. The phenomenon occurred frequently in paediatric patients and it was not possible to use BIPAP in patients with an endotracheal tube < 6 mm ID. The mode had to be changed to IPPV, but an inspiratory oscillatory flow still occurred in the IPPV when the pressure limit was active. We adjusted the V_T _carefully to avoid an oscillatory flow, on the one hand, and to avoid low minute ventilation, on the other.

You would not expect the P_aw _to be higher than that set on the ventilator in a pressure-regulated or pressure-limited mode, but exactly this happens when an oscillatory flow occurs. Unfortunately we have not measured the pressures in the test lung, but the following points led to our conclusion that the phenomenon is potentially dangerous and harmful. There is an airway pressure overshoot, the mean airway pressure is increased and the peak airway pressure may reach values above 50 cmH_2_O. The oscillations led to a jerky inspiration, showing that the pressure spikes really do reach the lung. Finally the pressure limit of the Oxylog 3000™ does not protect against the pressure overshoot.

The phenomenon of oscillatory inspiratory flow may impose as a malfunction of the device but it actually reflects a kind of limitation, of which the user should be aware and know how to deal with. We informed Dräger Medical in The Netherlands about our experiences and the results of the bench study. In the meantime, Dräger Medical started their own measurements and confirmed the validity of the problem. An adaptation of the operator's manual of the Oxylog 3000™ seems necessary, especially because the Oxylog 3000™ is licensed for ventilation with a very low V_T _(≥ 50 ml).

### Limitations

Spirometry was not obtained by a pneumotachograph, but with a spirometry module normally used for clinical purpose. This may especially affect the measurements of the compliance and the resistance, particularly in the tests with an oscillatory flow. Nevertheless, the flow curves and pressure curves obtained with this device were of good quality, and the results of the V_T _and other parameters were in accordance with the values displayed by the ventilators.

## Conclusion

Under conditions with high resistance an oscillatory inspiratory flow may occur during ventilation with the Oxylog 3000™ in the BIPAP, ASB, continuous positive airway pressure, and pressure-limited IPPV modes. The phenomenon results in elevated airway pressures and jerky inspiration. The unexpected high airway pressures may be potentially harmful, and therefore ventilation should be checked for the phenomenon in paediatric patients with narrow endotracheal tubes and in adult patients with severe obstructive airway or lung disease. If oscillations are present, the ventilator setting has to be adjusted by reducing the steepness of the ramp in BIPAP and ASB or by reducing the V_T _in pressure-limited IPPV.

## Key messages

• 	An oscillatory flow during inspiration may occur in pressure-regulated modes with the Oxylog 3000™, especially when airway resistance is high.

• 	The oscillatory flow results in a jerky inspiration and in elevated airway pressures.

• 	Peak airway pressures are markedly elevated above the set upper pressure limit and may cause lung injury.

## Abbreviations

ASB = assisted spontaneous breathing; BIPAP = biphasic intermittent positive airway pressure; F_I_O_2 _= fraction of inspired oxygen; I:E = inspiration to expiration time ratio; IPPV= intermittent positive pressure ventilation; P_aw _= airway pressure; P_aw-ampl _= amplitudes of the pressure oscillation; PEEP = positive end expiratory pressure; V_T _= tidal volume.

## Competing interests

The author(s) declare that they have no competing interests.

## Authors' contributions

GEF discovered the phenomenon under clinical conditions, designed the study, conducted the bench study, and analysed the results. HT assisted in designing the study and participated in interpreting the results. RDF performed statistical analysis and drafted the manuscript. All authors read and approved the final manuscript.

## Figures and Tables

**Table 1:** Course of the measurements including the settings of the lung simulator and the ventilators

Compliance, LS800™ (l/cmH_2_O)	Resistance, LS800™ (cmH_2_O/l/s)	Leak, LS800™	Endotracheal tube ID (mm)	HME type	D-fend™ type	Mode	F_I_O_2_	Respiratory rate (/min)	T_insp _(s)	I:E ratio	PEEP (cmH_2_O)	P_insp _(cmH_2_O)	Ramp, Oxylog 3000™	V_T _(ml)	Number of measurements, Oxylog 3000™	Comparable measurements, Evita-4 NeoFlow™
0.010	2, 4, 8, 16, 32, 64, 128	No	4	Baby	Paed	BIPAP	0.4	20	1.5	1/1	5	20	Slow std fast		21	15
0.007	2, 32, 64, 128	No	4	Baby	Paed	BIPAP	0.4	30	1	1/1	5	20	Slow std fast		12	4
0.015	2, 32, 64, 128	No	4	baby	Paed	BIPAP	0.4	20	1.5	1/1	5	20	Std		4	4
0.020	2, 32, 64, 128	No	5	Baby	Paed	BIPAP	0.4	20	1.5	1/1	5	20	Slow std fast		12	4
0.020	32	No	5	Baby	Paed	IPPV	0.4	20	1.5	1/1	5			180 – 500	8	0
0.020	2, 8, 32, 64, 128	No	7	Adult	Adult	BIPAP	0.8	20	1.5	1/1	15	35	Slow std fast		15	5
0.075	2, 8, 16, 32, 64, 128	No	7	Adult	Adult	BIPAP	0.8	16	1.9	1/1	10	28	Slow std fast		18	6
0.020	2, 8, 16, 32	No	7	Adult	Adult	ASB	0.8				6	20	Slow std fast		12	0
0.020	2, 8, 16, 32	No	7	Adult	Adult	CPAP	0.5				6	6			4	0
0.020	2, 8, 16, 32	No	7	Adult	Adult	ASB	0.5				6	20	Slow std fast		12	4
0.030	2, 8, 16, 32, 64, 128	No	7	Adult	Adult	BIPAP	0.5	12	1.6	1/2	8	26	Slow std fast		18	6
0.020	2, 8, 16, 32, 64, 128	No	7	Adult	Adult	BIPAP	0.5	12	1.6	1/2	8	26	Slow std fast		18	6
0.020	2	no	5	Paed	Paed	BIPAP	0.5	20	1.2	1/1.5	5	20	Slow std fast		3	0
0.020	2	Yes	5	Paed	Paed	BIPAP	0.5	20	1.2	1/1.5	5	20	Slow std fast		3	0
0.020	2	no	5.5	Paed	Paed	BIPAP	0.5	20	1.2	1/1.5	5	20	Slow std fast		3	0
0.020	2	Yes	5.5	Paed	Paed	BIPAP	0.5	20	1.2	1/1.5	5	20	Slow std fast		3	0
0.020	2	No	6	Paed	Paed	BIPAP	0.5	20	1.2	1/1.5	5	20	Slow std fast		3	0
0.020	2	Yes	6	Paed	Paed	BIPAP	0.5	20	1.2	1/1.5	5	20	Slow std fast		3	0
0.020	2	Yes	5	Paed	Paed	BIPAP	0.5	20	1.2	1/1.5	5	20	Std		1	0
0.020	2	Yes	5	Paed	Paed	BIPAP	0.4, 0.6, 0.8, 1	20	1.2	1/1.5	5	20	Fast		4	0
0.020	2	Yes	5	Paed	Paed	BIPAP	0.4, 0.6, 0.8, 1	20	1.2	1/1.5	5	20	Std		4	0
0.020	2	Yes	5	Paed	Paed	IPPV	0.5	20	1.2	1/1.5	5			240 – 600	8	0
0.020	2	No	5	Paed	Paed	IPPV	0.5	20	1.2	1/1.5	5			180 – 600	9	0

Related test-series with changes in one or maximal two parameters are grouped in one row. Numbers are values of the set parameters and do not reflect measured results. ID, internal diameter; T_insp _= inspiration time; I:E = inspiration to expiration time ratio; PEEP = positive end expiratory pressure; P_insp_, = set inspiratory airway pressure; V_T _= tidal volume; Paed, paediatric; std, standard; BIPAP = biphasic intermittent positive airway pressure; IPPV = intermittent positive pressure ventilation; ASB = assisted spontaneous breathing; CPAP, continuous positive airway pressure;

**Table 2:** Comparison between measurements showing oscillatory inspiratory flow andcorresponding Evita-4 NeoFlow™ measurements.

Parameter	*n*	Median		*P *value
			
		Oxylog 3000™	Evita-4 NeoFlow™	
Peak airway pressure (cmH_2_O)	40	32.65	20.8	<0.001
Mean airway pressure (cmH_2_O)	40	13.05	12.1	0.007
Expiratory tidal volume (ml)	40	133.05	136.35	0.840
Peak inspiratory flow (l/s)	40	16.05	11.85	0.023

Only tests with biphasic intermittent positive airway pressure and without leakage were included.

**Table 3:** Severity of the phenomenon

Parameter	Median	Interquartile range	Minimum	Maximum	*n*
Pressure amplitude (cmH_2_O)	12.15	17.75	0.10	37.9	153
Flow amplitude (l/s)	13.35	27.33	0.10	63.8	153
Airway pressure overshoot (cmH_2_O)	6.6	12.53	0.10	27.6	128
